# Effects of water-fertiliser coupling on the photosynthesis and quality of *Lycium barbarum* based on predicted crop evapotranspiration (ET_c_)

**DOI:** 10.1038/s41598-024-82986-4

**Published:** 2024-12-28

**Authors:** Yunfeng Liang, Dongpu Feng, Zhaojun Sun, Ping Ye, Shengfan Liang, Taiyue Shi

**Affiliations:** 1 School of Civil and Hydraulic Engineering, Ningxia University, Yinchuan, Ningxia, 750021 China; 2 School of Mechanical Engineering, Ningxia University, Yinchuan, Ningxia, 750021 China; 3 Engineering Research Center for Efficient Utilization of Modern Agricultural Water Resources in Arid Regions, Ministry of Education, Ningxia University, Yinchuan, 750021 Ningxia China; 4 School of Geography and Planning, Ningxia University, Yinchuan, Ningxia, 750021 Ningxia China; 5 China-Arab Joint International Research Laboratory for Featured Resources and Environmental Governance in Arid Region, Yinchuan, Ningxia, 750021 China; 6 Key Laboratory of Resource Assessment and Environmental Control in Arid Region of Ningxia, Yinchuan, Ningxia, 750021 China; 7 Pengyang County Agricultural Comprehensive Development Service Center, Pengyang, Ningxia, 756500 China

**Keywords:** Predicted crop water requirements, Water-fertiliser coupling, *Lycium barbarum*, Photosynthesis, Quality, Agroecology, Hydrology, Plant physiology

## Abstract

*Lycium barbarum* is an important economic crop in the arid region of Northwest China, and the regulation of irrigation and fertilisation is an important way to improve the quality and yield of *Lycium barbarum*. To explore the effects of water-fertiliser coupling on photosynthesis, quality and yield of *Lycium barbarum* under irrigation methods based on predicted crop evapotranspiration (ET_c_), ET_c_ was calculated via reference evapotranspiration (ET_o_) predicted on the basis of public weather forecasts, and the irrigation water volume was determined as a proportion of this ET_c_. A field experiment was conducted via a completely randomised experimental design with five irrigation water volumes (W0 (100% ET_c_), W1 (90% ET_c_), W2 (80% ET_c_), W3 (70% ET_c_) and W4 (65% ET_c_)) and three fertiliser application rates (high fertiliser (FH), medium fertiliser (FM) and low fertiliser (FL)). The results revealed that the chlorophyll content, G_sw_, C_i_ of *Lycium barbarum* leaves and 100-grain weight and yield of fresh fruit of *Lycium barbarum* increased with increasing irrigation, and the protein content, fat content, total sugar content and polysaccharide content in the dried fruits of *Lycium barbarum* first increased and then decreased with increasing irrigation under the same level of fertilisation, and the maximum value of these indexes reached 70% ET_c_ − 100% ET_c_. At the same irrigation level, E, A, G_sw_, C_i_ of *Lycium barbarum* leaves and protein content, fat content, total sugar content and polysaccharide content in the dried fruits of *Lycium barbarum* increased and then decreased with increasing of fertiliser application volume, and these indexes reached the maximum value at the fertiliser application rate of FM. A comprehensive evaluation based on principal component analysis (PCA) revealed that the optimum treatment in both years was W0FM (irrigation level of 100% ET_c_, corresponding to irrigation water in the range of 254.2–309.4 mm, and fertiliser application of N-P_2_O_5_-K_2_O of 315-82-135 kg ha^−1^), which was significantly greater in 2021 (2022) than in the CK. E, A, G_sw_ and C_i_ of daily changes of *Lycium barbarum* leaves in the W0FM treatment in 2021 (2022) increased by 46.54% (31.53%), 7.08% (59.26%), 18.55% (10.74%) and 34.58% (29.81%), respectively. In 2021 (2022), W0FM treatment increased fat content, polysaccharide content, and betaine content of dried fruits of *Lycium barbarum* and the 100-grain weight and yield of fresh fruit by 2.88% (10.11%), 1.56% (10.02%), 8.37% (21.69%), 13.57% (24.81%) and 31.39% (71.50%), respectively. The results of this study may provide a theoretical basis for improving the quality and efficiency of *Lycium barbarum* in the field in the arid zone of Northwest China.

*Lycium barbarum* is an important economic crop in arid and semiarid regions of China, both as a fruit and as a traditional Chinese medicine. It is rich in many nutrients, such as protein, fat, sugar and betaine. Ningxia, China, is the main production area with the best quality of *Lycium barbarum*^1^. However, Ningxia is dry with little rain and strong evaporation, and farmers and enterprises often invest excessive water and fertiliser in pursuit of high yields. However, excessive water and fertiliser inputs not only reduce the quality and yield of *Lycium barbarum*^2^but also lead to a decrease in soil quality and even cause deterioration of the farmland environment^3^. Therefore, it is necessary to explore reasonable water and fertiliser management strategies to improve the quality and yield of *Lycium barbarum* in arid and semiarid areas.

Moisture is necessary for plants to carry out their physiological activities and soil water is the main source of water uptake by plants. Therefore, regulating soil moisture through irrigation can affect crop photosynthesis and, consequently, fruit quality. Proper irrigation can improve crop quality and increase yield. Wang et al.^4^ reported that high soil moisture content had a significant negative effect on the vitamin, organic acid and carbohydrate contents of *Lycium barbarum* fruits. Consequently, the quality of the dried fruits of *Lycium barbarum*was greater under the deficit irrigation (gradual reduction in irrigation until irrigation was reduced by 50%) regime than under the control irrigation (100% plant evapotranspiration) regime^2^. Zhang et al.^5^ showed that the yield of dried fruits of *Lycium barbarum* was significantly greater at irrigation rates of 220–315 mm through water treatments based on the soil matric potential. Like irrigation, proper fertilisation can increase the yield of *Lycium barbarum*^6^ and improve *Lycium barbarum*quality^7^. However, overfertilisation can cause the quality of *Lycium barbarum* to decrease or even reduce yield, such as overfertilisation with nitrogen, which leads to a decrease in *Lycium barbarum*polysaccharides^8–10^, and overfertilisation with phosphorus, which decreases both the yield of *Lycium barbarum* and polysaccharides content of *Lycium barbarum*^11^. Different levels of water and fertiliser have significant combined effects on crop growth, quality and yield^12^. Studies have shown that an irrigation quota of 2,565 m^3^ ha^−1^ and a nitrogen application rate of 225 kg ha^−1^ are the best water and nitrogen management modes for drip-irrigated *Lycium barbarum* in the central arid zone of Ningxia on the basis of a comprehensive evaluation of photosynthesis, quality, and yield indicators of *Lycium barbarum*^13,14^. The combination of irrigation levels of 65–75% field holding capacity (279.38 mm and 315.41 mm in two years, respectively) and N application of 300 kg ha^−1^ was the best water and nitrogen regulation model for the photosynthesis, quality and yield of *Lycium barbarum*in the Yellow River Irrigation Zone of Gansu^15–17^. However, these studies on *Lycium barbarum*irrigation regimes^1,18^, *Lycium barbarum*nitrogen application^9–11^, *Lycium barbarum*phosphorus application^19^, *Lycium barbarum*nitrogen and phosphorus application^6^, and *Lycium barbarum*water-nitrogen coupling^13,14^ have been conducted in the form of irrigation quotas or *Lycium barbarum*irrigation regimes through upper and lower soil moisture limit controls determined as a percentage of field water holding capacity^20–22^ and *Lycium barbarum*water-nitrogen coupling^15–17,23^ studies. These irrigation regimes, obtained from irrigation quota methods or upper and lower soil moisture limit studies, have a certain lag, which makes it difficult to ensure the quality and yield of Lycium barbarum in arid and semiarid areas.

In fact, the water required by a crop consists of plant transpiration and interplant soil evaporation, and the total amount of water consumed by the two is called crop evapotranspiration (ET_c_)^24^. Therefore, in recent years, some scholars have proposed irrigation methods based on ET_c_, such as irrigation with different percentages of ET_c_, which has been widely used as an experimental factor in water-fertiliser coupling experiments for crops such as sunflower^25,26^, seed cotton^27^, mango^28–30^and potato^31–33^. Since ET_c_-based irrigation methods consider meteorological factors and changes in the water requirements of crops at different fertility stages, they play a role in precise regulation of soil moisture, fertiliser and the environment during irrigation and thus have good effects on crop photosynthesis^31–33^, quality^28–33^and yield^25–33^. However, the ET_c_used in these studies were calculated via historical measured meteorological data and empirical formulas^25–33^. This kind of irrigation using historical ET_c_ to decide is not sufficient considering the future uncertainty. After irrigation based on historical ET_c_, if there is a short-term rainfall (sudden drought) in the future, it may cause too much (too little) soil moisture, resulting in flooding of the crop root system (drought), which may lead to a decrease in yield and quality. Therefore, some scholars have utilized future weather forecast data instead of historically measured meteorological data in real-time scheduling of irrigation. For example, short-term future weather forecast data and models have been used for irrigation management of maize crops^34–36^, lettuce irrigation regime optimization^37^, and rice irrigation decision-making^38^. The above studies have shown that various models or methods for irrigation decision making based on weather forecasts can effectively cope with drought^35,36^, improve rainfall utilization^38^and maintain stable crop yields^34–38^. However, there is a lack of research on the use of future weather forecast data for irrigation scheduling in crop quality regulation.

Therefore, the hypothesis of this study was that the optimal regulation of soil water-fertiliser coupling under irrigation, as determined on the basis of the predicted ET_c_, would enhance the ability of *Lycium barbarum* plants to carry out photosynthesis and improve the quality of *Lycium barbarum*. The objectives of this study were as follows: (1) to explore the effects of the combination of water and fertiliser on the photosynthesis and quality of *Lycium barbarum* under irrigation on the basis of predicted ET_c_ and (2) to use a comprehensive method for evaluating the photosynthesis, quality and yield of *Lycium barbarum* and to determine the optimal water-fertiliser combinations under irrigation on the basis of the predicted ET_c_ to achieve relatively high-quality *Lycium barbarum* in arid zones of China.

## Materials and methods

### Experimental site

**Fig. 1:**
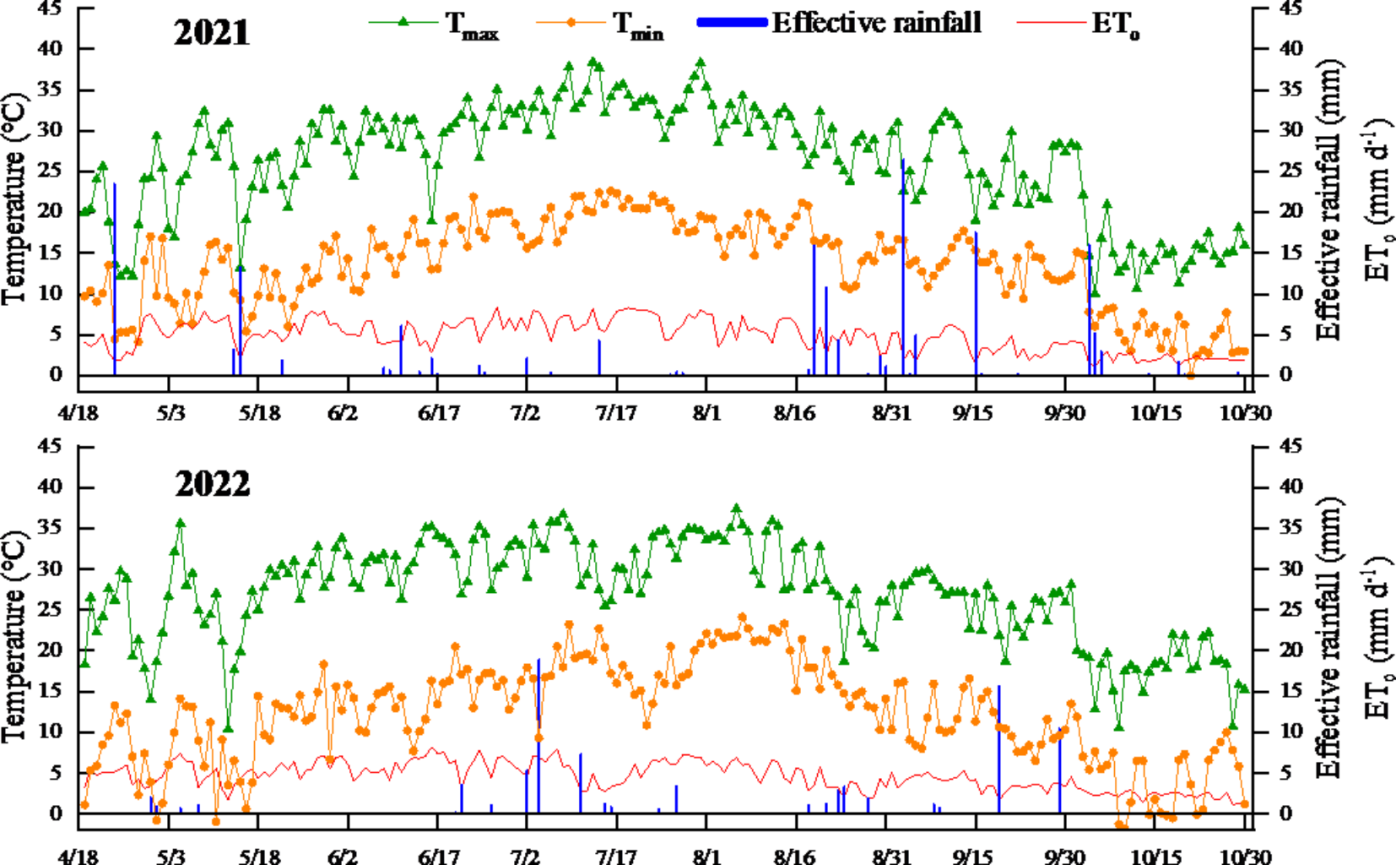
Daily trends in reference evapotranspiration (ET_o_) and meteorological variables for the test period in 2021 (2022).

A field experiment was conducted from April 2021 to October 2022 in Tongde village, Hexi town, Tongxin County, Wuzhong city, Ningxia, China (latitude 37°9′58″N, longitude 105°43′19″E). The experimental site belongs to the semiarid zone of the mesothermal zone, with a multiyear average precipitation of 272.6 mm, a multiyear average temperature of 8.6 ℃, and a multiyear average evapotranspiration of 2,387.0 mm. Meteorological data from the experimental field were monitored in real time by a portable meteorological station (Farmland Meteorological Station Vantage Pro2, Davis, U.S.A.) installed near the experimental area. The meteorological parameters and reference evapotranspiration (ET_o_) during the experimental period are shown in Fig. 1.

### Experimental design

In this experiment, under drip irrigation conditions, a completely randomised experimental design was used and included two factors, irrigation and fertiliser application, where five irrigation water amounts (W0 (100% ET_c_), W1 (90% ET_c_), W2 (80% ET_c_), W3 (70% ET_c_), and W4 (65% ET_c_), ET_c_ is the predicted crop evapotranspiration), and three fertiliser rates (high fertiliser (FH): N-P_2_O_5_-K_2_O of 420-109-180 kg ha^−1^; medium fertiliser (FM): N-P_2_O_5_-K_2_O of 315-82-135 kg ha^−1^; low fertiliser (FL): N-P_2_O_5_-K_2_O of 200-55-90 kg ha^−1^) were used. One control (CK) was set as follows: irrigation water, 247 mm; fertiliser application, N-P_2_O_5_-K_2_O, 390-105-165 kg ha^−1 39^. The completely randomised experimental design for the two factors of irrigation amount and fertiliser amount is shown in Table 1. A total of 16 treatments were used in this experiment, and each treatment was replicated three times.

**Table 1:** The experimental design was completely randomised for two factors: the irrigation amount and the fertiliser amount.

Treatment	Irrigation amount (mm)	Fertiliser amount (*N*-*P*_2_O_5_-K_2_O) ( kg ha^−1^)
W0FH	100% ET_c_	420-109-180
W0FM	100% ET_c_	315-82-135
W0FL	100% ET_c_	200-55-90
W1FH	90% ET_c_	420-109-180
W1FM	90% ET_c_	315-82-135
W1FL	90% ET_c_	200-55-90
W2FH	80% ET_c_	420-109-180
W2FM	80% ET_c_	315-82-135
W2FL	80% ET_c_	200-55-90
W3FH	70% ET_c_	420-109-180
W3FM	70% ET_c_	315-82-135
W3FL	70% ET_c_	200-55-90
W4FH	65% ET_c_	420-109-180
W4FM	65% ET_c_	315-82-135
W4FL	65% ET_c_	200-55-90
CK	247	390-105-165

### Experimental implementation

The seedling variety of *Lycium barbarum*used for testing was 8-year-old ‘Ningqi No.7’, and the seedling variety selected for this study were provided by the Ningxia Runjia Agricultural and Forestry Development Co., Ltd., China. The row spacing was 3 m, plant spacing was 0.75 m. It was arranged in accordance with 1 treatment per row (row length of 15.75 m), and each treatment had 3 replications, for a total of 48 experimental plots. The area of each plot was 15.75 m^2^ (length, 5.25 m; width, 3 m; and 7 *Lycium barbarum*plants per plot). The experimental area was 708.75 m^2^. To ensure that the experimental area was free from the influence of the surrounding cultivation, 3 rows of protection rows were set up as isolation protection for the experimental area. The irrigation method is hedge frame drip irrigation, and the irrigator is an internal inlaid drip irrigation pipe with 16 mm diameter, a rated flow rate of 4 L h^−1^ (working flow rate of 2–3 L h^−1^), and a drip head spacing of 0.6 m.

The soil of the test field was clay with 32.03% (volumetric water content) water holding capacity. The soil bulk density was 1.62 g cm^−3^. The initial soil chemical properties in the 0–60 cm layer were as follows: total salt, 2.97 g kg^−1^; pH, 8.44; organic matter, 7.05 k kg^−1^; alkali nitrogen (N), 34.23 mg kg^−1^; soil available potassium (K), 199 mg kg^−1^; and soil available phosphorus (P) 7.05 mg kg^−1^. Fresh soil (0–60 cm) was collected prior to the experiment, air-dried to determine soil nutrients, and fertiliser application rates were determined via the nutrient balance method. The nitrogen, phosphorus and potash fertilisers selected for fertilization were urea (*N* ≥ 46.0%), monoammonium phosphate (12–60-0 for N-P_2_O_5_-K_2_O) and fully water-soluble potassium sulphate (K_2_O ≥ 52%), respectively, and fertiliser was applied via water-fertiliser integration, with six fertiliser applications (2021 and 2022) throughout the whole growth period. The specific fertiliser management practices used during the 2021 (2022) trial are shown in Table 2. Agronomic measures such as uniform pruning, hoeing, soil tillage and pest control were carried out in all treatments.

**Table 2:** Specific fertiliser management programme for the trial period in 2021 (2022).

Fertility stages	2021: Fertiliser amount (*N*-*P*_2_O_5_-K_2_) / kg ha^−1^				2022: Fertiliser amount (*N*-*P*_2_O_5_-K_2_) / kg ha^−1^			
	Data	FH	FM	FL	Data	FH	FM	FL
Budding period	04–16	85-13-20	64-10-15	41-7-10	04–15	85-13-20	64-10-15	41-7-10
Spring branch growth period	05–09	85-13-20	64-10-15	41-7-10	05–06	85-13-20	64-10-15	41-7-10
Flowering and fruit setting period	05–30	49-16-28	36-12-21	23-8-14	05–27	49-16-28	36-12-21	23-8-14
Fruit ripening period	06–14	49-16-28	36-12-21	23-8-14	06–11	49-16-28	36-12-21	23-8-14
	06–29	49-16-28	36-12-21	23-8-14	06–26	49-16-28	36-12-21	23-8-14
	07–14	49-16-28	36-12-21	23-8-14	07–11	49-16-28	36-12-21	23-8-14
Defoliation period	08–17	54-19-28	43-14-21	26-9-14	08–19	54-19-28	43-14-21	26-9-14
Total		420-109-180	315-82-135	200-55-90		420-109-180	315-82-135	200-55-90

In accordance with the local experience of *Lycium barbarum*cultivation, all plots were irrigated once each during the budding period and dormant period, with irrigation volumes of 525 m^3^ ha^−1^and 600 m^3^ ha^−1^, respectively. The irrigation experiment was implemented from mid-April 2021 (2022), with irrigation decisions made at intervals of 3 days, 53 times during the growth period of 2021 (and 62 times during the growth period of 2022).

### Methodology for irrigation decision making

In this study, public weather forecast data with a lead time of 1–3 days, the temperature Penman‒Monteith equation (PMT) empirical equation, and the integrated learning model gradient boosting with categorical features support (CatBoost) were used to predict daily ET_o_ with a lead time of 1–3 days. ET_c_ was subsequently calculated via the single crop coefficient method, and finally, irrigation decisions were made on the basis of the water balance equation combined with precipitation with a lead time of 1–3 days.

(1) Daily ET_o_ prediction method for a lead time of 1–3 days.

On the basis of the results of previous studies, the PMT method is more accurate from 19 April to 5 May and from 7 August to 30 October, so the PMT method is used in this period. The calculation procedure is as follows (1)–(10) Eq. 2^4^: The values of different parameters in the empirical equation of the PMT are corrected via the trial-and-error method to minimise the root-mean-square error, and after the correction, the daily ET_o_ can be predicted via using the daily maximum and daily minimum temperatures as inputs to the PMT Eq. 4^0^. In spring, the PMT empirical equations predicted daily ET_o_ for a lead time of 1–3 days with a mean absolute error (MAE) of 0.883 mm d^−1^, a root mean square error (RMSE) of 1.149 mm d^−1^, and a correlation coefficient (R) of 0.608; in autumn, the PMT empirical equations predicted daily ET_o_ for a lead time of 1–3 days with a mean MAE of 0.968 mm d^−1^ and a mean RMSE of 1.285 mm d^−1^with an average R of 0.598^40^.

1 $$\:\begin{array}{c}{\text{E}\text{T}}_{\text{o}}=\frac{0.408\varDelta\:\left({R}_{n}-G\right)+\gamma\:\frac{900}{T+273}{u}_{2}\left({e}_{s}-{e}_{a}\right)}{\varDelta\:+\gamma\:\left(1+0.34{u}_{2}\right)}\end{array}$$

2 $$\:\begin{array}{c}{e}_{s}=\frac{{e}^{o}\left({T}_{max}\right)+{e}^{o}\left({T}_{min}\right)}{2}=\frac{0.611{exp}\left[\frac{17.27{T}_{max}}{{T}_{max}+237.3}\right]+0.611{exp}\left[\frac{17.27{T}_{min}}{{T}_{min}+237.3}\right]}{2}\end{array}$$

3 $$\:\begin{array}{c}{e}_{a}={e}^{o}\left({T}_{dew}\right)=0.611{exp}\left[\frac{17.27{T}_{dew}}{{T}_{dew}+237.3}\right]\end{array}$$

4 $$\:\begin{array}{c}{R}_{a}=\frac{24\left(60\right)}{\pi\:}{G}_{sc}{d}_{r}\left[{\omega\:}_{s}{sin}\left(\phi\:\right){sin}\left(\delta\:\right)+{cos}\left(\phi\:\right){cos}\left(\delta\:\right){sin}\left({\omega\:}_{s}\right)\right]\end{array}$$

5 $$\:\begin{array}{c}{R}_{s}={k}_{Rs}\sqrt{\left({T}_{max}-{T}_{min}\right)}{\times\:R}_{a}\end{array}$$

6 $$\:\begin{array}{c}{R}_{so}=\left({a}_{s}+{b}_{s}\right){R}_{a}\end{array}$$

7 $$\:\begin{array}{c}{R}_{ns}=\left(1-\alpha\:\right){R}_{s}\end{array}$$

8 $$\:\begin{array}{c}{R}_{nl}=\sigma\:\left[\frac{{{T}_{max,K}}^{4}+{{T}_{min,K}}^{4}}{2}\right]\left(<span class='convertEndash'>0.34-0.14</span>\sqrt{{e}_{a}}\right)\left(1.35\frac{{R}_{s}}{{R}_{SO}-0.35}\right)\end{array}$$

9 $$\:\begin{array}{c}{R}_{n}={R}_{ns}-{R}_{nl}\end{array}$$

10 $$\:\begin{array}{c}{u}_{2}=\frac{4.87{u}_{z}}{ln(67.8z-5.42)}\end{array}$$

where ET_o_ is the daily reference evapotranspiration [mm day ^−1^]; R_n_ is the net radiation at the crop surface [MJ m^−2^ day^−1^]; G is the soil heat flux density [MJ m^−2^ day^−1^], and G may be ignored for daily periods; T is the mean daily air temperature at 2 m height [°C]; u_2_ is the wind speed at 2 m height [m s^−1^]; e_s_ is the saturation vapour pressure [kPa]; e_a_ is the actual vapour pressure [kPa]; es -ea is the saturation vapour pressure deficit [kPa]; ∆ is the slope vapour pressure curve [kPa °C^−1^]; γ is the psychrometric constant [kPa °C^−1^]; e^o^ (T_max_) is the saturation vapour pressure at daily maximum temperature [kPa]; e^o^ (T_min_) is the saturation vapour pressure at daily minimum temperature [kPa]; T_max_ and T_min_ are the daily maximum and daily minimum air temperature, respectively [℃]; R_a_ is the extraterrestrial radiation [MJ m^−2^ day^−1^]; G_SC_ is a solar constant = 0.0820 [MJ m^−2^ min^−1^]; d_r_ is the inverse relative distance between the Earth and Sun; _s_ is the sunset hour angle [rad]; is the latitude [rad]; δ is the solar declination [rad]; R_s_ is solar or shortwave radiation [MJ m^−2^ day^−1^]; k_Rs_ is the adjustment coefficient (0.16.0.19) [℃^−0.5^], and for ‘interior’ locations k_Rs_=0.16, for ‘coastal’ locations k_Rs_=0.19; as + b_s_ is the fraction of extraterrestrial radiation reaching the earth on clear-sky days (n = N), Where no actual solar radiation data are available and no calibration has been carried out for improved a_s_ and b_s_ parameters, the values as = 0.25 and b_s_ = 0.50 are recommended; R_so_ is the clear-sky solar radiation [MJ m^−2^ day^−1^]; R_ns_ is the net solar or shortwave radiation [MJ m^−2^ day ^−1^]; α is albedo or canopy reflection coefficient (α = 0.23); R_nl_ is the net outgoing longwave radiation [MJ m ^−2^ day ^−1^]; σ is Stefan-Boltzmann constant [4.903 10 ^−9^ MJ K ^−4^ m ^−2^ day ^−1^]; T_max, K_ is the maximum absolute temperature during the 24-hour period [K = °C + 273.16]; T_min, K_ is the minimum absolute temperature during the 24-hour period [K = °C + 273.16]; uz is the wind speed at z m height [m s^−1^]; z is the station elevation above sea level [m].

The CatBoost model is more accurate from 5 May to 7 August, and the prediction of daily ET_o_ can be achieved by using the daily maximum temperature, daily minimum temperature, and Wspd (converting the wind levels in the public weather forecast to wind speed (Wspd) according to Table 3) from public weather forecasts with a lead time of 1–3 days as the inputs to CatBoost. In summer, the Catboost model predicted daily ET_o_ for a lead time of 1–3 days with a mean MAE of 1.368 mm d^−1^, and the mean RMSE was 1.670 mm d^−1^with an average R of 0.169^40^.

**Table 3:** Beaufort wind scale (GB/T 35227—2017, 2017).

Wind scale	Designation	u_10_(m s^−1^)	
		Range	Average
0	Calm	0.0–0.2	0.0
1	Light	0.3–1.5	1.0
2	Slight	1.6–3.3	2.0
3	Gentle	3.4–5.4	4.0
4	Moderate	5.5–7.9	7.0
5	Fresh	8.0–10.7	9.0
6	Strong wind	10.8–13.8	12.0
7	High wind	13.9–17.1	16.0
8	Gale	17.2–20.7	19.0
9	Strong gale	20.8–24.4	23.0
10	Whole gale	24.5–28.4	26.0
11	Storm	28.5–32.6	31.0
12	Hurricane	32.7–36.9	35.0

(2) ET_c_ is calculated as follows:

11 $$\:{ET}_{c}={K}_{c}{ET}_{o}$$

The daily ET_o_ was determined according to the method of prediction in (1); the crop coefficient K_c_ of *Lycium barbarum*was 0.77 (budding period), 0.96 (spring branch growth period), 0.96 (flowering and fruit setting period), 0.8 (fruit ripening period), and 0.69 (defoliation period)^41^.

**Fig. 2:**
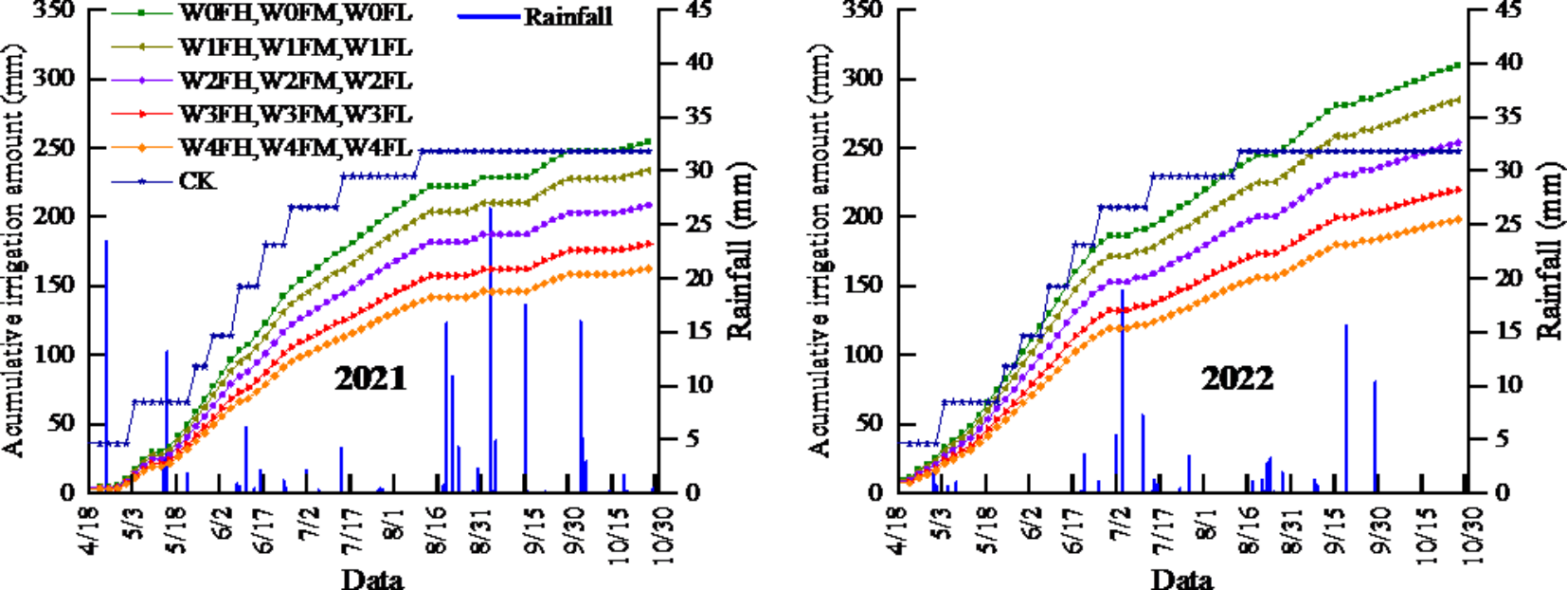
Cumulative irrigation for different treatments during the experiment (19 April to 30 October).

(3) In this study, the difference between the sum of ET_c_ for a 1–3 day lead time and the sum of effective rainfall for a 1–3 day lead time was used as the amount of water per irrigation, as determined by Eq. (12).

12 $$\:M=\beta\:\times\:\left[\sum\:_{i=1}^{3}\left({{K}_{c}ET}_{o\left(t+i\right)}\right)-\sum\:_{i=1}^{3}\left({\alpha\:P}_{\left(t+i\right)}\right)\right]\times\:{r}_{s}\times\:{p}_{s}\times\:n\times\:{r}_{n}\times\:{W}_{r}\times\:{10}^{-9}$$

where ET_c_ is the crop evapotranspiration (mm); K_c_ is the crop coefficient; ET_o_ is the reference evapotranspiration (mm); M denotes the sum of ET_c_for a 1–3-day lead time (m^3^); ET_o(t+1)_, ET_o(t+2)_, and ET_o(t+3)_ are the predicted reference evapotranspiration (mm) based on the public weather forecast with a 1-day lead time (t + 1), 2-day lead time (t + 2), and 3-day lead time (t + 3), respectively (mm); P_(t+1)_, P _(t+2)_ and P _(t+3)_ are the amount of rainfall (mm) monitored by the meteorological stations during the periods t + 1, t + 2 and t + 3 days within the 1–3 days lead time, respectively; and α is the rainfall infiltration coefficient (the value of α is related to the intensity of each rainfall event, the magnitude of the rainfall event, the duration of the rainfall event, the nature of the soil, the ground cover, and the topography of the terrain, among other factors). The values are shown in Table 4^42^; r_s_ is the row spacing of *Lycium barbarum* plants (mm); p_s_ is the spacing of *Lycium barbarum* plants (mm); n is the number of *Lycium barbarum* plants in a row; r_n_ is the number of rows; W_r_ is the drip irrigation soil wetting ratio (%), which refers to the percentage of soil volume wetted by the drip irrigation scheme to the total soil volume of the wet layer of the irrigation scheme, which is often expressed as the percentage of the total irrigated area that is wet at 20–30 cm below the ground surface; and W_r_ is 50 for *Lycium barbarum*^39^. The cumulative irrigation amounts for the 16 treatments during the 2021 (2022) trial period are shown in Fig. 2.

**Table 4:** The values of the rainfall infiltration coefficient α (in arid zones, when rainfall amount is < 5 mm, α = 1.).

Rainfall amount (mm)	< 5	5–50	> 50
α	0*	0.8–1.0	0.7–0.8

### Measurements and methods

Irrigation water amount: The total irrigation water volume was monitored and read by an electromagnetic flow metre (BLD-DN50, China, Kaifeng Hexin Instrument Co., Ltd.), and the irrigation water volume of each test treatment was monitored and read by a rotary-wing wet-type water metre (LXS-DN40CE, China, Linyi Jinhai Instrument Manufacturing Co., Ltd.).

Soil water content: A soil moisture sensor (MAS-1 Professional Current, METER, USA) calibrated by the drying method was used to monitor the volumetric water content of the soil in the four layers of 0–20 cm, 20–40 cm, 40–60 cm and 60–80 cm in real time, and the locations of the four layers of soil moisture sensors were 10 cm, 30 cm, 50 cm and 70 cm, respectively.

The photosynthesis of *Lycium barbarum* leaves was measured every 2 h from 08:00 a.m. to 6:00 p.m. on sunny and less cloudy days via a portable photosynthetic fluorescence measurement system (LI-6800, USA, LI-COR Inc.), with three plants per treatment repeated each time. These indicators included transpiration (E, mol m^−2^ s^−1^), net photosynthetic rate (A, µmol m^−2^ s^−1^), stomatal conductance (G_sw_, mol m^−2^ s^−1^), and intercellular carbon dioxide concentration (C_i_, µmol mol^−1^) of the leaves of *Lycium barbarum* plants. Photosynthesis was measured three times throughout the growth period.

A chlorophyll meter (SPAD-502 Plus, Japan, Konica Minolta) was used to measure the chlorophyll content of the labelled leaves. Three *Lycium barbarum* plants were measured per treatment, and three labelled leaves were measured per *Lycium barbarum* plant.

Soil nutrients: Soil samples were collected from three soil layers, 0–20 cm, 20–40 cm and 40–60 cm, for each treatment before the experiment to determine the initial nutrients of the soil. The soil pH, soil total salts, soil available potassium (K), soil organic matter, soil alkaline nitrogen (N) and soil available phosphorus (P) content were determined via potentiometric, conductometric, flame photometric, potassium dichromate volumetric-external heating, alkaline dissolved diffusion, and 0.05 mol-L^−1^ NaHCO_3_methods, respectively^43^.

Fruit quality indicators: The protein content of the dried fruit of *Lycium barbarum*was determined via the combustion method^44^; the fat content of the dried fruit of *Lycium barbarum*was determined via the Soxhlet extraction method^45^; the polysaccharide and total sugar contents of the dried fruit of *Lycium barbarum*were both determined via the standard method^46^; and the betaine content of the dried fruit of *Lycium barbarum*was determined by high-performance liquid chromatography^47^.

Yield: All the *Lycium barbarum* plants in each treatment group were harvested, and the fresh fruit yield of the *Lycium barbarum* plants in each treatment group was the sum of the fresh fruit yield of the *Lycium barbarum* plants at each harvest.

### Data processing

Excel 2016 software was used for preliminary data processing, and SPSS 22.0 software (IBM Corporation, Armonk, NY, USA) and Data Processing System 18.10 (Hangzhou Ruifeng Information Technology Co., Ltd.) software were used to perform analysis of variance (ANOVA) and Duncan’s multiple comparisons of the observed data of *Lycium barbarum*. Principal component analysis (PCA) was used for comprehensive evaluation^33,48,49^. Origin 2021 learning version software and Origin 2024 learning version software (OriginLab Inc., USA) were used for darwing.

(1) Standardisation of raw indicator data from the tests

13 $$\:\begin{array}{c}{x}_{ij}^{*}=\frac{{x}_{ij}-{\stackrel{-}{x}}_{j}}{{s}_{j}}\:\:\:(i=1,\:2,\dots\:,n;\:j=1,\:2,\dots\:,m;)\end{array}$$

where m denotes the number of indicators in the experimental treatment; n denotes the number of observations for each experimental indicator; $\:{\stackrel{-}{x}}_{j}=\frac{1}{n}\sum\:_{i=1}^{n}{x}_{ij}$; $\:{s}_{j}=\sqrt{\frac{1}{n-1}\sum\:_{i=1}^{n}{\left({x}_{ij}-{\stackrel{-}{x}}_{j}\right)}^{2}}$.

(2) Calculate the correlation coefficient matrix

14 $$\:R={\left({r}_{ij}\right)}_{m\times\:m}$$

15 $$\:\begin{array}{c}{r}_{ij}=\frac{\sum\:_{i=1}^{n}({x}_{ki}-{\stackrel{-}{x}}_{i})\left({x}_{kj}-{\stackrel{-}{x}}_{j}\right)}{\sqrt{\sum\:_{i=1}^{n}{{\left({x}_{ki}-{\stackrel{-}{x}}_{i}\right)}^{2}\left({x}_{kj}-{\stackrel{-}{x}}_{j}\right)}^{2}}}\end{array}$$

where $\:{r}_{ij}$=1 and $\:{r}_{ij}$=${r}_{ji};{r}_{ij}$ is the correlation coefficient between the ith test indicator and the jth test indicator.

(3) Calculate the eigenvalues and eigenvectors of R

16 $$\mid\lambda\:E-R\mid=0$$

where λ is the eigenvalue and R is the correlation coefficient matrix. The eigenvalues are sorted according to $\:{\lambda\:}_{1}$≥$\:{\lambda\:}_{2}$≥…≥$\:{\lambda\:}_{m}$≥0, the corresponding eigenvectors $\:{\alpha\:}_{i}$(i=1, 2, ……)are found, and $\:\sum\:_{j=1}^{m}{\alpha\:}_{ij}=1$, $\parallel{\alpha}_{i}\parallel=1$.

(4) Determining the principal components and calculating the composite score.

First, the information contribution rate and cumulative contribution rate of the eigenvalues are calculated via Eqs. (17) and (18), and then the composite score is calculated via Eq. (19).

17 $$\:\begin{array}{c}{C}_{j}=\frac{{\lambda\:}_{j}}{\sum\:_{k=1}^{m}{\lambda\:}_{k}}\end{array}$$

18 $$\:\begin{array}{c}A{C}_{P}=\frac{\sum\:_{k=1}^{p}{\lambda\:}_{k}}{\sum\:_{k=1}^{m}{\lambda\:}_{k}}\end{array}$$

19 $$\:\begin{array}{c}Z=\sum\:_{j=1}^{p}{C}_{j}{y}_{j}\end{array}$$

where y_j_ denotes the jth principal component (j = 1, 2…, m); C_j_ is the information contribution rate of the principal component yj; AC_p_ is the cumulative contribution rate of the principal components y_1_, y_2_, …, y_p_; and Z is the composite score of the jth principal component.

## Results and analyses

### Effects of different water-fertiliser combinations on the photosynthesis of *Lycium barbarum* plants on the basis of the predicted ET_c_

**Fig. 3:**
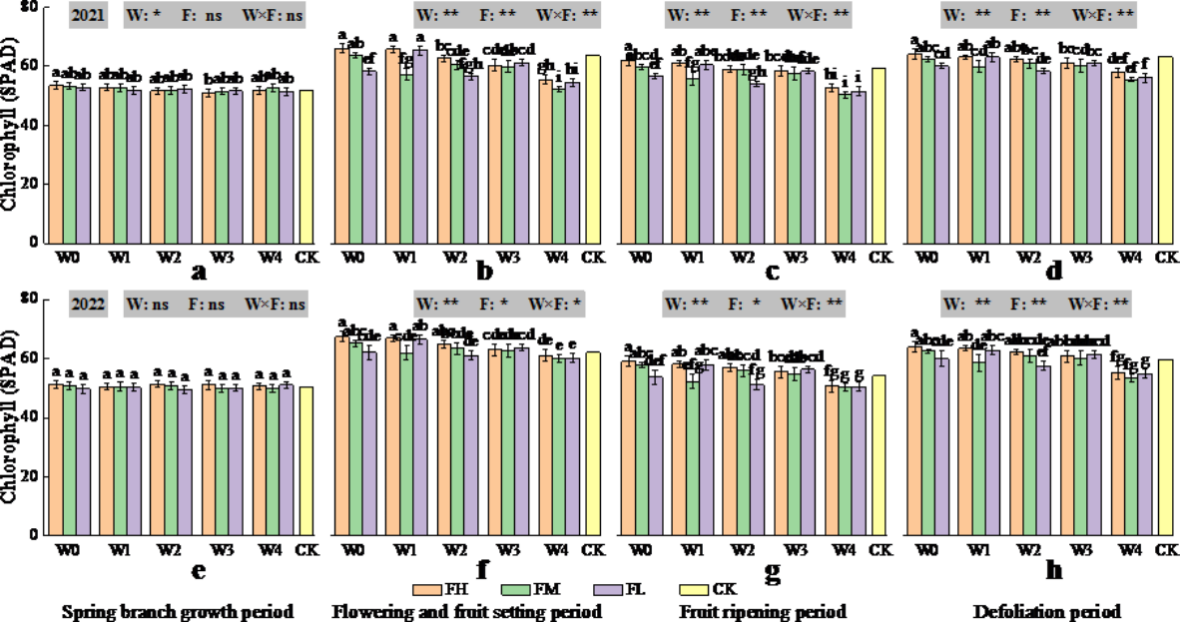
Changes in the chlorophyll content of *Lycium barbarum* ((**a**) Spring branch growth period, (**b**) Flowering and fruit setting period, (**c**) Fruit ripening period and (**d**) Defoliation period in 2021 and (**e**) Spring branch growth period, (**f**) Flowering and fruit setting period, (**g**) Fruit ripening period and (**h**) Defoliation period in 2022) after different water and fertiliser treatments (W indicates the watering level, F indicates the fertiliser level, CK indicates the experimental control, where * and ** indicate significant differences at the *P* < 0.05 and *P* < 0.01 levels, respectively, and ns indicates no significant difference (*P* > 0.05). Different symbols above the error bars indicate significant differences between treatments according to Duncan’s multiple range test (*P* < 0.05).).

In 2021 (2022), the chlorophyll content of *Lycium barbarum* leaves showed a similar pattern of change during the two growth periods of *Lycium barbarum*, i.e., from the spring branch growth stage to the flowering and fruit setting stage gradually increased, the fruit ripening period slightly decreased, and the defoliation period gradually increased (Fig. 3). In the two growth periods of *Lycium barbarum* in 2021 (2022), the chlorophyll content of W0FH treatment in the flowering and fruit setting stages was the highest. In terms of the chlorophyll content at the defoliation stage, the effect of irrigation volume on the basis of the predicted ET_c_ on the chlorophyll content was highly significant (*P* < 0.01) compared with that in the CK; for both W0-W1, the chlorophyll content increased; for W2–W3, the change in chlorophyll content was not significant, whereas for W4, the change in chlorophyll content decreased. The effect of fertiliser application on the chlorophyll content was highly significant (*P* < 0.01). Compared with the CK, in W0, FH increased the chlorophyll content, FM did not significantly affect the chlorophyll content, and FL decreased the chlorophyll content. The interaction of irrigation and fertiliser application had a highly significant (*P* < 0.01) effect on the chlorophyll content. Compared with the CK treatment, in 2021 (2022), W0FH resulted in the maximum chlorophyll content, which increased by 1.39% (7.09%), whereas W4FM resulted in the minimum chlorophyll content, which decreased by 12.13% (10.14%).

**Fig. 4:**
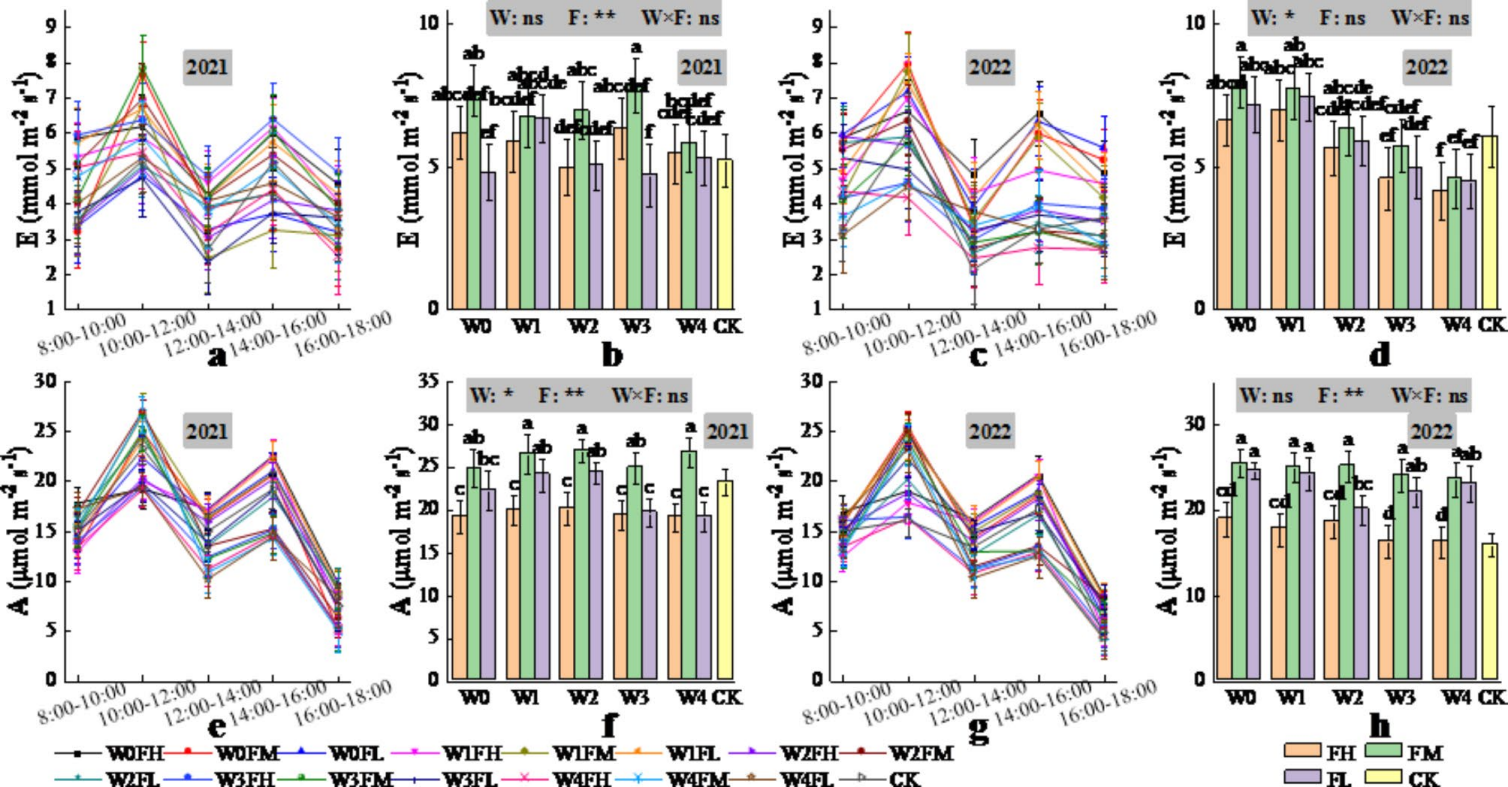
Daily changes in E (2021 (**a**), 2022 (**c**)) and A (2021 (**e**), 2022 (**g**)) of *Lycium barbarum* under different water and fertilisation treatments over time (data were obtained from the observation data of *Lycium barbarum* during the flowering and fruit setting stages: observation data of 4 June 2021 and 1 June 2022. The vertical line in the graph indicates the standard deviation), and changes in E (2021 (**b**), 2022 (**d**)) and A (2021 (**f**), 2022 (**h**)) of *Lycium barbarum* under different water and fertilisation treatments (The data analysed here for E and A are for the period 10:00–12:00. W indicates the watering level, F indicates the fertiliser level, CK indicates the experimental control, where * and ** indicate significant differences at the *P* < 0.05 and *P* < 0.01 levels, respectively, and ns indicates no significant difference (*P* > 0.05). Different symbols above the error bars indicate significant differences between treatments according to Duncan’s multiple range test (*P* < 0.05).).

**Fig. 5:**
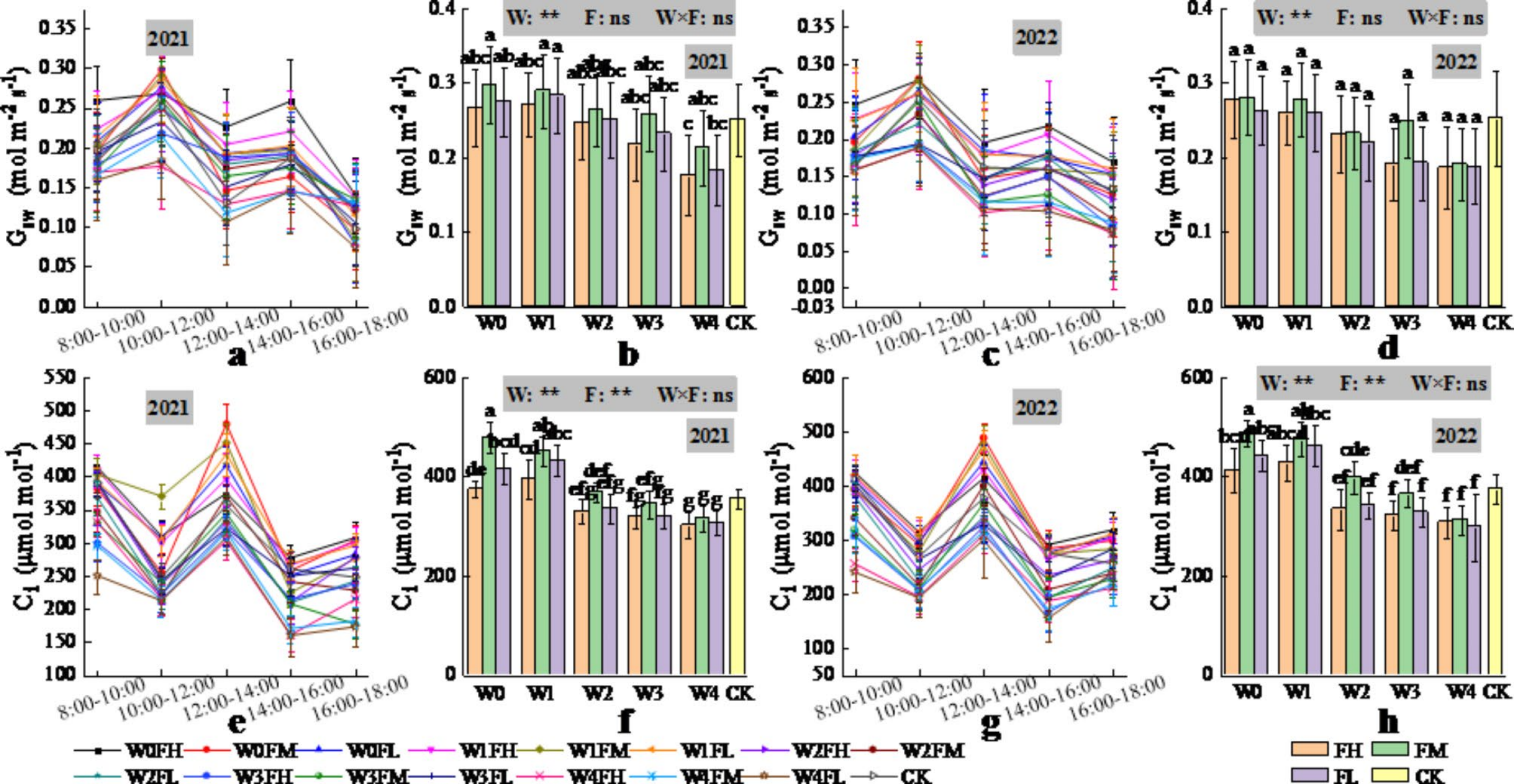
Daily changes in G_sw_ (2021 (**a**), 2022 (**c**)) and C_i_ (2021 (**e**), 2022 (**g**)) of *Lycium barbarum* under different water and fertilisation treatments over time (data were obtained from the observation data of *Lycium barbarum* during the flowering and fruit setting stages: observation data of 4 June 2021 and 1 June 2022. The vertical line in the graph indicates the standard deviation), and changes in G_sw_ (2021 (**b**), 2022 (**d**)) and C_i_ (2021 (**f**), 2022 (**h**)) of *Lycium barbarum* under different water and fertilisation treatments (the data analysed here for G_sw_ is for the period 10:00–12:00 and those for C_i_ are for the period 12:00–14:00. W indicates the watering level, F indicates the fertiliser level, CK indicates the experimental control, where * and ** indicate significant differences at the *P* < 0.05 and *P* < 0.01 levels, respectively, and ns indicates no significant difference (*P* > 0.05). Different symbols above the error bars indicate significant differences between treatments according to Duncan’s multiple range test (*P* < 0.05).).

In both years, the daily trends of transpiration (E, mol m^−2^ s^−1^), net photosynthetic rate (A, µmol m^−2^ s^−1^) and stomatal conductance (G_sw_, mol m^−2^ s^−1^) of the *Lycium barbarum* leaves were all “M” shaped, with peaks occurring from 10:00–12:00 and from 14:00–16:00, respectively; the highest peaks occurred from 10:00–12:00; the daily trend of intercellular carbon dioxide concentration (C_i_, µmol mol^−1^) of the *Lycium barbarum* leaves was the “W” type; and the highest peaks occurred from 12:00–14:00 (Figs. 4 and 5).

In 2021, fertiliser application had a highly significant (*P* < 0.01) effect on E (Fig. 4), but irrigation volume, which was determined on the basis of the predicted ET_c,_ and the interaction of irrigation and fertiliser application had a nonsignificant (*P* > 0.05) effect on E. Compared with CK, FM increased E, FH had a nonsignificant change in E, and FL decreased E. In 2022, the irrigation volume, which was determined on the basis of the predicted ET_c,_ had a significant (*P* < 0.05) effect on E, compared with CK, W0 and W1 increased E, W2 and W3 had insignificant changes in E, and W4 decreased E. However, fertiliser application and the interaction of irrigation and fertiliser application did not have a significant (*P* > 0.05) effect on E. Maximum E was achieved by W3FM (W0FH), with a 50.49% (30.40%) increase in 2021 (2022) compared with CK.

The irrigation volume determined on the basis of the predicted ET_c_ had a significant (*P* < 0.05) effect on A in 2021 (Fig. 4), compared with CK, with W1 and W2 increasing A and W0 having a nonsignificant change in A and W3-W4 decreasing A. Fertiliser application in both years had a highly significant (*P* < 0.01) effect on A, compared with the CK, with FM increasing A and FL having a nonsignificant change in A and FH decreasing A. The interaction effect of irrigation and fertiliser application had no significant (*P* > 0.05) effect on A in either year. In 2021 (2022), W2FM (W0FH) achieves the largest A compared to CK, increasing by 15.98% (59.12%).

The irrigation volume determined on the basis of the predicted ET_c_ had highly significant (*P* < 0.01) effects on both G_sw_ and C_i_ (Fig. 5), compared with the CK, W0 and W1 increased G_sw_ and C_i_, W2 had insignificant effects on G_sw_ and C_i_, and W3 and W4 decreased G_sw_ and C_i_. Fertiliser application had highly significant (*P* < 0.01) effects on C_i_ and nonsignificant (*P* > 0.05) effects on G_sw_. Compared with the CK treatment, FM increased C_i_, whereas FL did not significantly affect C_i_, and FH decreased C_i_. The interaction effect of irrigation and fertiliser application had nonsignificant (*P* > 0.05) effects on G_sw_ and C_i_. Compared with CK, W0FH achieves the largest G_sw_ and C_i_ in 2021 (2022), increasing by 69.20% (10.67%) and 34.58% (29.81%), respectively.

In conclusion, the results of the two-year study revealed that the chlorophyll content, G_sw_ and C_i_ of the different water and fertiliser treatments increased with increasing irrigation in 2021 (2022), and E, A, G_sw_, and C_i_ increased and then decreased with increasing fertiliser application. The W0FM treatment promoted photosynthesis of *Lycium barbarum* plants and facilitated the accumulation of dry matter in *Lycium barbarum* plants.

### Effects of different water-fertiliser combinations on the quality of the dried fruits of *Lycium barbarum* based on the predicted ET_c_

**Fig. 6:**
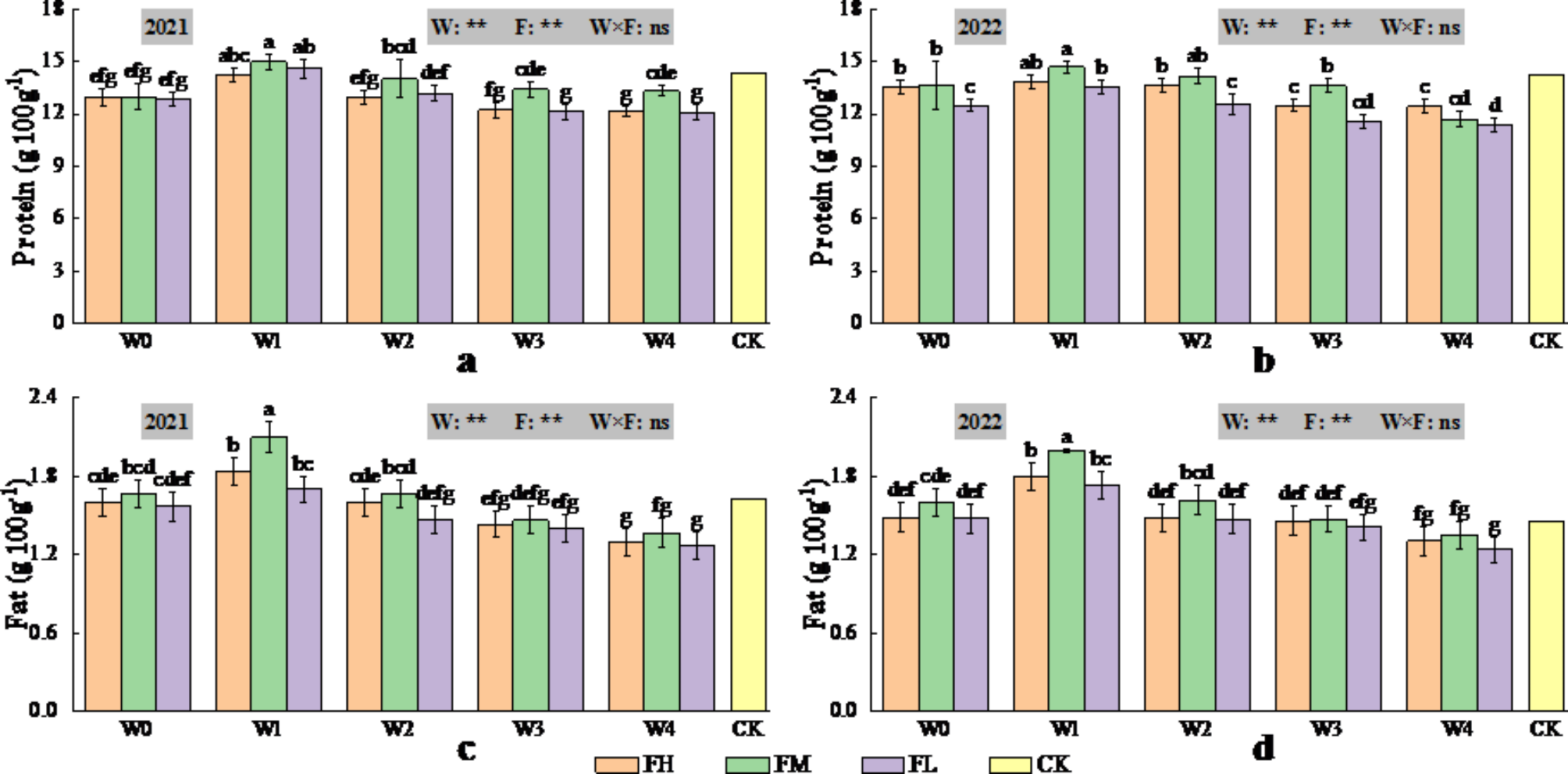
Changes in protein content (2021 (**a**) Protein, 2022 (**b**) Protein) and fat content (2021 (**c**) Fat, 2022 (**d**) Fat) in dried fruits of *Lycium barbarum* under different water and fertilisation treatments (W indicates the watering level, F indicates the fertiliser level, CK indicates the experimental control, where * and ** indicate significant differences at the *P* < 0.05 and *P* < 0.01 levels, respectively, and ns indicates no significant difference (*P* > 0.05). Different symbols above the error bars indicate significant differences between treatments according to Duncan’s multiple range test (*P* < 0.05).).

**Fig. 7:**
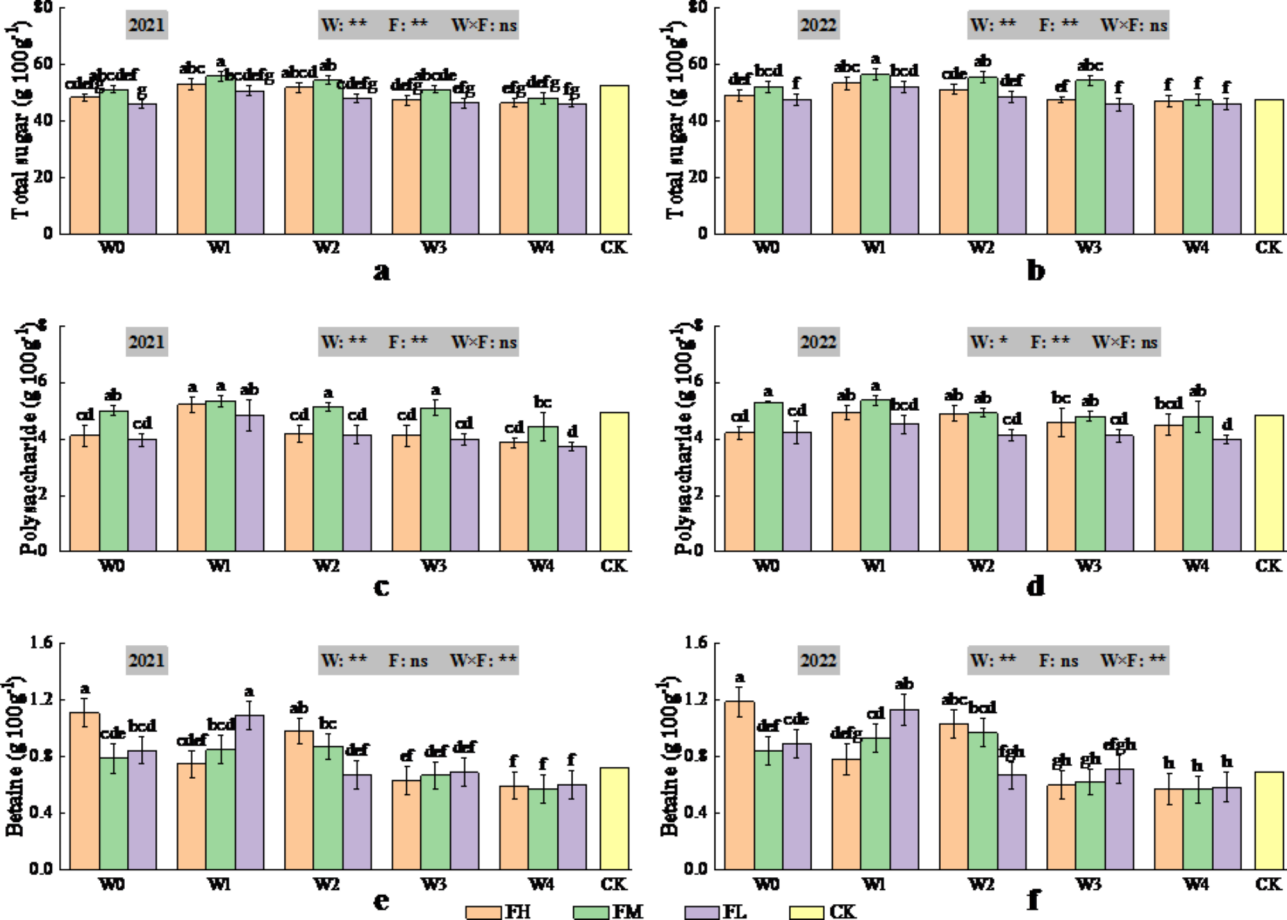
Changes in total sugar content (2021 (**a**) Total sugar, 2022 (**b**) Total sugar), polysaccharide content (2021 (**c**) Polysaccharide, 2022 (**d**) Polysaccharide) and betaine content (2021 (**e**) Betaine, 2022 (**f**) Betaine) of dried fruits of *Lycium barbarum* under different water and fertilisation treatments (W indicates the watering level, F indicates the fertiliser level, CK indicates the experimental control, where * and ** indicate significant differences at the *P* < 0.05 and *P* < 0.01 levels, respectively, and ns indicates no significant difference (*P* > 0.05). Different symbols above the error bars indicate significant differences between treatments according to Duncan’s multiple range test (*P* < 0.05).).

The effects of irrigation volume determined on the basis of the predicted ET_c_ were highly significant (*P* < 0.01) for protein, fat, total sugars and polysaccharides (Figs. 6 and 7) and significant (*P* < 0.05) for polysaccharides in 2021 (Fig. 7), with protein, fat, total sugars and polysaccharides all increasing and then decreasing with increasing irrigation volume, all of which reached their maximum values at W1. The effects of fertiliser application on protein, fat, total sugar and polysaccharide contents were highly significant (*P* < 0.01), compared with those of the CK treatment, and for W1, protein, fat, total sugar and polysaccharide contents all increased and then decreased with increasing fertiliser application, all of which reached their maximum values at FM. The interaction effect of irrigation and fertiliser application did not significantly (*P* > 0.05) affect protein, fat, total sugars or polysaccharides.

The irrigation volume determined on the basis of the predicted ET_c_ had a highly significant (*P* < 0.01) effect on betaine (Fig. 7), and compared with the CK treatment, W0-W2 increased betaine, W3 did not significantly change betaine, and W4 decreased betaine. However, fertiliser application did not significantly (*P* > 0.05) affect betaine. The interaction of irrigation and fertiliser application had a highly significant (*P* < 0.01) effect on betaine. Compared with the CK, W0FH achieved a maximum betaine content, with a 52.88% (71.86%) increase in 2021 (2022), whereas W4FM achieved a minimum betaine content, with a 20.74% (17.60%) decrease.

**Fig. 8:**
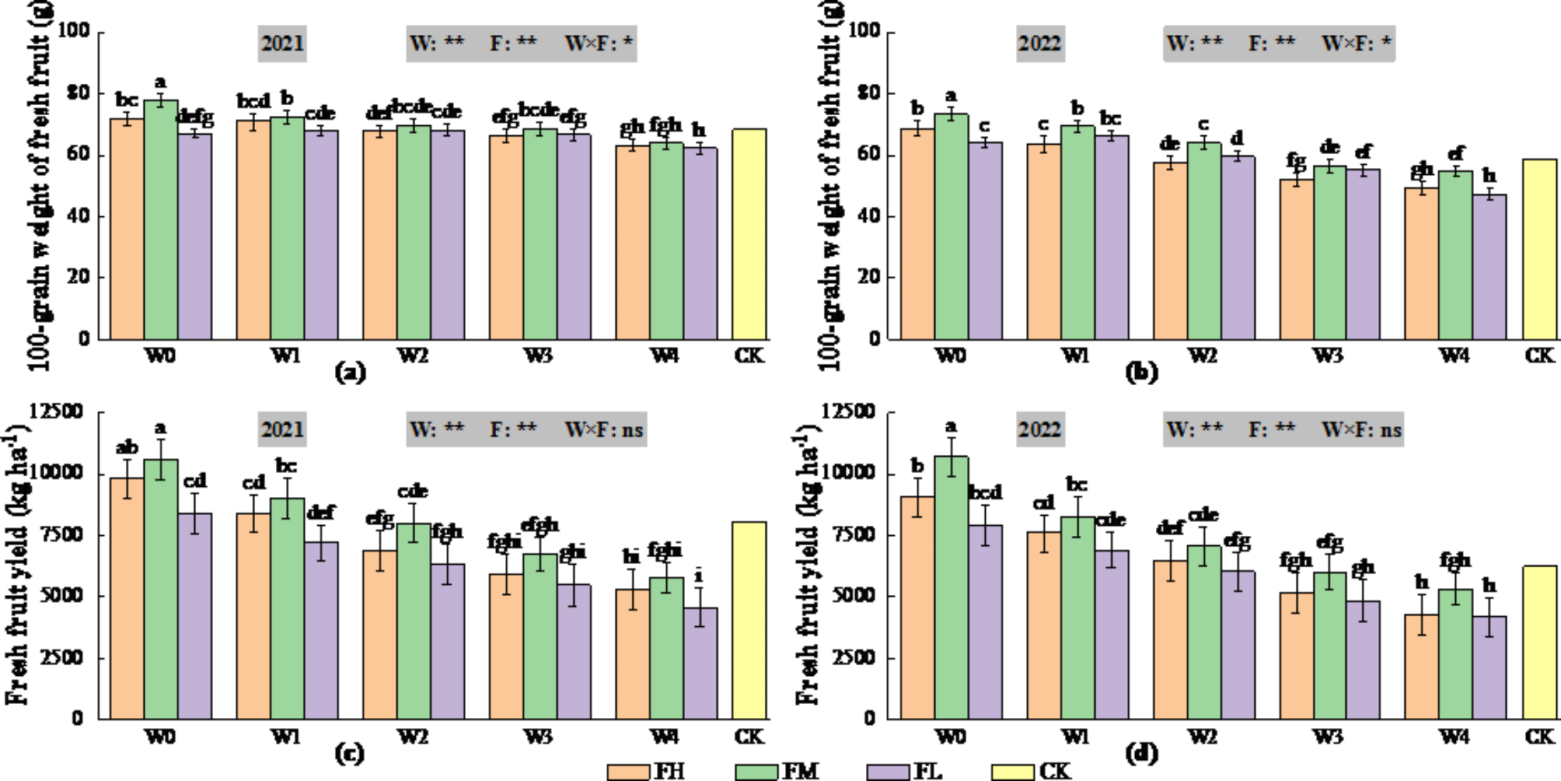
Changes in the 100-grain weight of fresh fruit of *Lycium barbarum* (2021 (**a**) 100-grain weight of fresh fruit, 2022 (**b**) 100-grain weight of fresh fruit) and fresh fruits yield of *Lycium barbarum* (2021 (**c**) Fresh fruits yield, 2022 (**d**) Fresh fruits yield) under different water and fertilisation treatments (W indicates the watering level, F indicates the fertiliser level, CK indicates the experimental control, where * and ** indicate significant differences at the *P* < 0.05 and *P* < 0.01 levels, respectively, and ns indicates no significant difference (*P* > 0.05). Different symbols above the error bars indicate significant differences between treatments according to Duncan’s multiple range test (*P* < 0.05).).

Irrigation volume determined based on the predicted ETc had highly significant (*P* < 0.01) effects on yield and 100-grain weight (Fig. 8). Compared with CK, W0 increased yield, and the changes in yield were not significant in W1 and W2, whereas W3 and W4 reduced yield. Compared with CK, W0 and W1 increased 100-grain weight, and the changes in 100-grain weight were not significant in W2 and W3, whereas W4 decreased 100-grain weight. The effect of fertilizer application was highly significant (*P* < 0.01) on both yield and 100-grain weight, and compared with CK, for W0, FH and FM increased yield and 100-grain weight, while the change in yield and 100-grain weight was not significant for FL. The interaction of irrigation and fertilizer application had a significant (*P* < 0.05) effect on 100-grain weight and a non-significant (*P* > 0.05) effect on yield. Compared with CK, W0FM resulted in the greatest yield and 100-grain weight in 2021 (2022), increasing by 31.39% (71.50%) and 13.57% (24.81%), respectively, whereas W4FL resulted in the smallest yield and 100-grain weight, decreasing by 43.38% (32.96%) and 8.80% (19.56%), respectively.

In conclusion, the results of the two-year study revealed that the water-fertiliser combination of W1FM was more conducive to increasing the protein content, fat content, total sugar content and polysaccharide content of dried fruits, whereas the water-fertiliser combination of W0FH was more conducive to increasing the betaine content of dried fruits, and the water-fertiliser combination of W0FM was more conducive to increasing the yield of fresh fruit and the 100-grain weight of fresh fruit.

### Integrated evaluation based on PCA

Since the optimal water-fertilisation combinations considering physiological indexes and the quality and yield of *Lycium barbarum* differ, a comprehensive analysis is needed.

(1) Correlation analysis between experimental indicators.

**Fig. 9:**
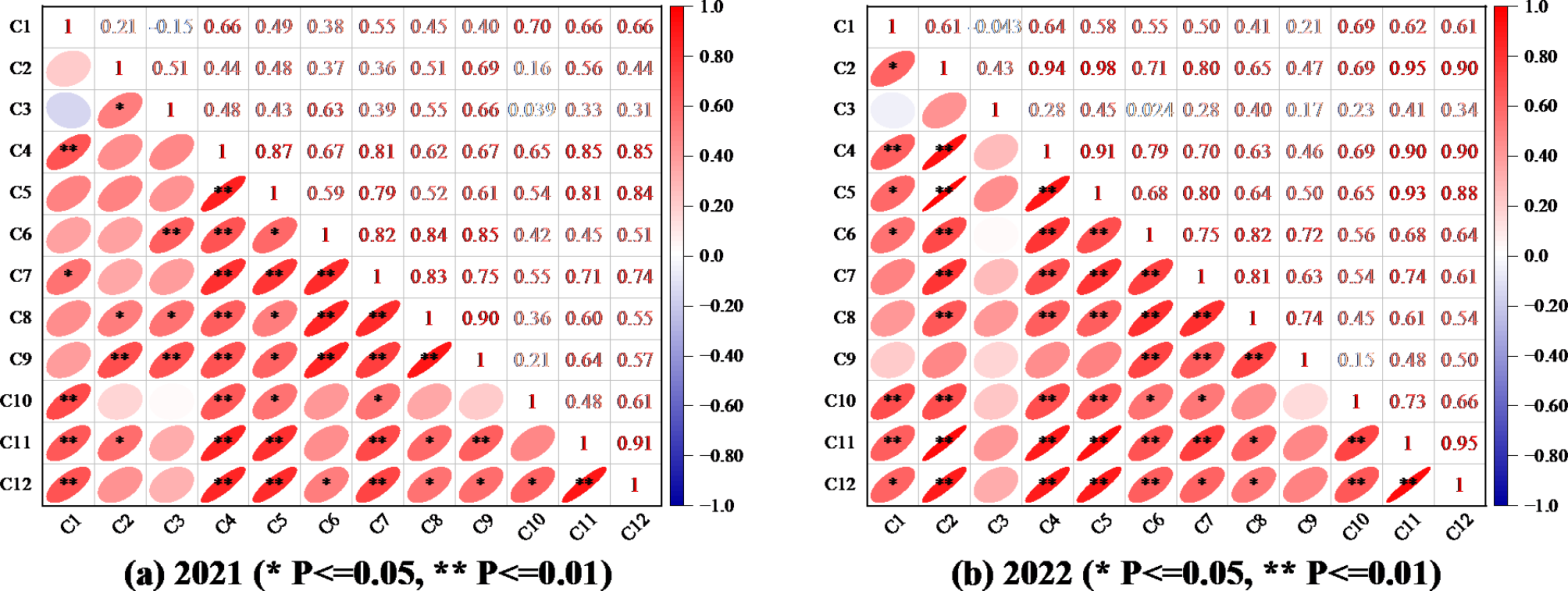
Heat map of correlation between test indicators in 2021 (2022) (C1 for chlorophyll, C2 for E, C3 for A, C4 for G_sw_, C5 for C_i_, and C6 for protein, C7 for fat, C8 for total sugar, C9 for polysaccharide, C10 for betaine, C11 for 100-grain weight of fresh fruit, C12 for fresh fruit yield).

Figure 9 shows that the yield of fresh fruit of *Lycium barbarum* in 2021 (2022) was different degrees of correlation with chlorophyll, E, A, G_sw_, C_i_, protein, fat, total sugar, polysaccharide, betaine, and fresh fruit 100-grain weight, and the direct comprehensive evaluation produces repeated information and affects the evaluation results; therefore, the physiological indexes of *Lycium barbarum* (chlorophyll, E, A, G_sw_, C_i_), quality (protein, fat, total sugar, polysaccharide, betaine) and yield (fresh fruit 100-grain weight), all of which have different correlations with the fresh fruit yield of *Lycium barbarum*, were selected as evaluation indexes, and PCA was applied to comprehensively evaluate each experimental treatment.

(2) Comprehensive evaluation based on PCA.

As shown in Table 5, the cumulative contribution rate of the eigenvalues of the first three principal components in 2021 was 85.01% (greater than 80%); the cumulative contribution rate of the eigenvalues of the first three principal components in 2022 was 87.17% (greater than 80%), which indicated that the three extracted principal components were able to represent 85.01% (87.17%) of the information of the original 10 experimental indicators.

**Table 5:** Contruibution rate and cumulative contribution rate of principal compoinents.

Time	Principal component	Eigenvalues	Contribution rate	Cumulative contribution rate
2021	pc1	7.41	61.78	61.78
	pc2	1.87	15.55	77.33
	Pc3	0.92	7.68	85.01
2022	pc1	7.94	66.17	66.17
	pc2	1.34	11.21	77.37
	Pc3	1.18	9.80	87.17

The principal components for 2021 (pc4-pc10) and 2022 (pc4-pc10) are not shown in the table because they contain only 14.01% and 13.21% of the original variance information.

The total score for each treatment was calculated via Eq. (19) and is shown in Table 6. Therefore, the optimal treatment for 2021 (2022) was W0FM. Compared with those in the CK treatment, the fat, polysaccharides, betaine, fresh fruit the 100-grain weight, and fresh fruit yields of *Lycium barbarum* in the W0FM treatment in 2021 (2022) increased by 2.88% (10.11%), 1.56% (10.02%), 8.37% (21.69%), 13.57% (24.81%) and 31.39% (71.50%), respectively. Among the other treatments, the medium fertilizer treatment was ranked higher for the same irrigation amount, and the treatment with a high irrigation amount was ranked higher for the same fertilization rate. The rankings of the optimum results were consistent in both years, and the other ranking results were slightly different but not much different, indicating that the method of water and fertilizer regulation was relatively stable and reliable.

**Table 6:** Comprehensive evaluation of the effects of water-fertiliser coupling on physiological indicators, quality and yield of *Lycium barbarum* via PCA.

Treatment	2021					2022				
	PC1	PC2	PC3	Total score	Ranking	PC1	PC2	PC3	Total score	Ranking
W0FH	1.15	−2.90	0.50	0.32	4	2.05	−2.51	−0.95	1.07	6
W0FM	3.70	−0.38	2.64	2.65	1	4.06	−0.04	0.98	3.03	1
W0FL	−0.41	−1.38	0.23	−0.49	10	0.89	−1.72	1.36	0.58	10
W1FH	2.26	−0.38	−0.91	1.38	5	2.26	0.51	−1.38	1.55	4
W1FM	4.44	1.43	−0.73	3.17	2	4.40	1.91	0.69	3.48	2
W1FL	2.49	−0.41	−0.70	1.55	3	2.72	−0.94	0.42	1.89	3
W2FH	−0.32	−1.47	−1.25	−0.57	8	0.18	0.06	−1.41	−0.01	7
W2FM	2.27	1.37	−0.23	1.75	6	1.92	0.91	0.38	1.54	5
W2FL	−1.34	0.52	−0.05	−0.82	12	−1.52	−0.44	0.25	−1.13	12
W3FH	−2.27	−0.34	0.63	−1.53	11	−2.61	0.31	−1.36	−1.99	11
W3FM	0.64	1.77	0.97	0.82	9	0.10	1.28	0.30	0.26	9
W3FL	−2.68	−1.07	−0.12	−1.99	13	−2.70	−1.18	0.35	−2.06	15
W4FH	−4.19	0.16	0.14	−2.78	14	−3.93	0.76	−0.78	−2.83	13
W4FM	−2.25	2.49	0.10	−1.09	15	−3.12	1.08	1.46	−1.96	14
W4FL	−4.62	0.41	0.09	−3.03	16	−4.49	−0.30	1.30	−3.14	16
CK	1.10	0.19	−1.30	0.66	7	−0.21	0.32	−1.63	−0.29	8

## Discussion

### Effects of different irrigation rates and fertiliser rates on various indicators of photosynthesis in *Lycium barbarum* plants

Chlorophyll is the key pigment that converts light energy into chemical energy during plant photosynthesis, and its content is a direct response to the photosynthetic performance of leaves, which is affected mainly by changes in the soil microclimate^50–54^. Studies have shown that increasing the amount of irrigation and fertiliser can increase the leaf nitrogen content, which in turn can increase the chlorophyll content of leaves^55^. In the present study, during the two *Lycium barbarum*growth periods in 2021 (2022), the chlorophyll content tended to increase, then decreased and then increased, which is consistent with the results of previous studies^14,17^. This occurred because the water and nutrients in the root zone of the crop are allocated mainly to the nutritive growth of *Lycium. barbarum* during the early growth period (spring branch growth period and flowering and fruit setting period), whereas in the late growth period (fruit ripening period), they are allocated mainly to the reproductive growth of *Lycium barbarum*, which leads to significant differences in chlorophyll content during the different fertility periods of *Lycium barbarum*^13^.

In addition, in this study, the daily variations in E, A, and G_sw_ were all “M” type, with peaks occurring sequentially in the 10:00–12:00 and 14:00–16:00 periods. This finding is consistent with the results of previous studies on crops such as *Lycium barbarum*^16^and grape^55^. This occurred because during the 8:00–10:00 am period, the light intensity and temperature were low, and the stomatal conductance of the plant leaves gradually increased, resulting in a low transpiration rate and net photosynthetic rate. As the light intensity and temperature increased, the stomatal resistance gradually increased, resulting in a decrease in stomatal conductance and a gradual increase in transpiration and photosynthetic rates, with the 1st peak occurring during the 10:00–12:00 period. As the light intensity and temperature continued to increase, the leaf temperature continued to rise, the relative humidity of the air decreased, and the leaf water deficit intensified, resulting in a rapid decrease in leaf stomatal conductance, the leaf transpiration rate and photosynthetic rate rapidly decreased. At 12:00–14:00, the leaf “lunch break” phenomenon gradually increased. At this time, because the stomata partially closed, the transpiration rate decreased, the amount of transpirational water loss decreased, the transpiration force forced the root system to absorb water to replenish the water in the leaves, the stomatal conductance gradually increased, the transpiration rate and photosynthesis rate slowly increased, and the second peak occurred at 14:00–16:00. However, the daily changes in C_i_ in this study were all “W” type, with the peak occurring in the 12:00–14:00 period. This was mainly due to the increase in A during the 10:00–12:00 and 14:00–16:00 periods, which consumed a large amount of CO_2_ and resulted in two troughs at approximately 10:00 and 14:00, respectively, whereas during the leaf ‘lunch break’, due to the closure of the leaf stomata, the intercellular CO_2_ concentration briefly accumulated, resulting in a peak at 12:00–14:00. A similar result was reported by Gao et al.^16^ for *Lycium barbarum*.

In addition, In the CK treatment, A in 2022 was 0.68 times greater than A in 2021, and the yield was 0.77 times greater. This occurred because precipitation decreased more in 2022, while the irrigation quota of the CK treatment was fixed, which led to water stress in the crop receiving water and a decline in some indicators of the crop. For the W0FL treatment, the E in 2022 was 1.49 times greater than the E in 2021, which could be due to several reasons. First, the irrigation rate of 100%- 80% ET_c_is a dynamic irrigation system, the dry climate in 2022 is coupled with sufficient water supply, so evaporation is high, and E in 2022 is generally higher than E in 2021. Second, the nutrient growth of the crop may be more vigorous than reproductive growth in the case of low fertilizer application, which leads to ineffective evapotranspiration on the high side^56^. Additionally, the fertilizer and irrigation treatments as well as the meteorological factors in the different years affected the phenological period of *Lycium barbarum*^57,58^, but the observation dates were relatively fixed, and the phenological period of the same treatment on the same date might have differed.

The results of the present study revealed that increasing irrigation increased E, A, G_sw_, and C_i_ in *Lycium barbarum* leaves; moreover, E, A, G_sw_, and C_i_ in *Lycium barbarum* leaves increased but then decreased with increasing fertiliser application at the same irrigation rate. This finding is similar to the results of previous studies on crops such as *Lycium barbarum*^16^and cotton^59^, which are attributed to the fact that moderate and large amounts of irrigation increase the chlorophyll content, which improves the photosynthetic capacity of the crop and enhances the E, A, G_sw_, and C_i_^60^ of the crop. However, under reduced irrigation, an insufficient water supply destroys the chlorophyll structure, which leads to the decomposition of pigmentation, decreased chlorophyll content, and decreased carbon dioxide solubility in the leaf saprophyll cells, and reduced the solubility of CO_2_in the chloroplasts, thereby reducing the photosynthetic rate of the crop^61^. However, high fertiliser levels force the accumulation of nitrate in the soil, which reduces the uptake capacity of the root system, leading to premature leaf ageing, blockage of CO_2_in the green leaves, and a weakening of the photosynthetic rate^62^.

### Effect of different irrigation volumes and fertiliser applications on the quality and yield of *Lycium barbarum*

Soil water is the medium for soil nutrient transformation and nutrient uptake by plant roots. Therefore, soil water is directly related to fruit quality. The results of this study revealed that the protein content, fat content, total sugar content and polysaccharide content of dried fruits first increased but then decreased with increasing irrigation volume, possibly because when the irrigation volume increased from W4 to W1, a reasonable water supply increased the normal water demand of *Lycium barbarum*, accelerated the activity of photosynthesis-related enzymes and chlorophyll synthesis, and promoted reactive oxygen metabolism in the crop, which had a positive impact on the quality of *Lycium barbarum*. However, when the irrigation amount increased from W1 to W0, the soil water content in the root zone of *Lycium barbarum* increased rapidly due to the large amount of irrigation, accelerating the transfer of phloem sap to *Lycium barbarum*. The increased water flow from the xylem to *Lycium barbarum* resulted in the dilution of the protein content, fat content, total sugar content and polysaccharide content per unit dry matter weight in *Lycium barbarum*^33^. Consequently, the increases in the protein content, fat content, total sugar content and polysaccharide content of the dried fruits were significantly reduced by increasing the irrigation volume from W1 to W0. This finding is similar to the results of previous studies on crops such as *Lycium barbarum*^2,13^, mango^29^, potato^33^and tomato^63^. In addition, Kong et al.^64^ reported that moderate nitrogen levels increased the soluble protein content in chilli, Song et al.^65^ reported that the starch and protein contents of potato tubers increased and then decreased with increasing nitrogen application under the same moisture conditions, and both Ma et al.^13^ and Gao et al.^16^ noted that the total sugar content, polysaccharide content, protein content and fat content of *Lycium barbarum* increased but then decreased with increasing nitrogen application. These findings are consistent with the results of the present study, i.e., the protein content, fat content, total sugar content and polysaccharide content of *Lycium barbarum* were highest at the FM fertiliser level. This may have occurred because when the fertiliser application increased from FL to FM, the soil fertility of the root system of *Lycium barbarum* improved, which promoted root growth and effectively delayed the ripening time of *Lycium barbarum*. Moreover, the photosynthesis time of the crop was prolonged, and the protein content, fat content, total sugar content and polysaccharide content of *Lycium barbarum* were also effectively improved. In addition, moderate application of fertiliser promoted the transformation of plant materials and the synthesis of organic macromolecules in storage organs and improved the quality *of Lycium barbarum*. However, when the fertiliser application increased from FM to FH, the soil nutrient content was significantly greater than the plant nutrient uptake, resulting in a large nutrient residue in the root zone and a high soil ion concentration per unit volume. This had a toxic effect on the root system of *Lycium barbarum* and reduced its quality. In conclusion, this study revealed that when mild water deficit (W2-W1) was combined with moderate fertiliser application (FM), the quality of *Lycium barbarum* improved, and the protein content, fat content, total sugar content and polysaccharide content of *Lycium barbarum* increased most significantly, which was mainly because the irrigation volume determined on the basis of the predicted ET_c_and fertiliser application provided suitable water and fertiliser inputs, which allowed the soil enzyme activities to be fully utilised and improved the soil microbial environment, which enhanced the absorption and regulation of soil nutrients (e.g., nitrogen, phosphorus, and potash) by the root system and effectively promoted the interaction of water and fertiliser, thus improving the quality^66–71^.

Irrigation is the main way to replenish soil moisture in field crops in western China, where drought and low rainfall are common. The effects of irrigation on plant growth and fruit quality depend on the timing and amount of irrigation. With the same total amount of irrigation (the irrigation amount of CK in 2021 is close to that of W0, and the irrigation amount of CK in 2022 is close to that of W2, as shown in Fig. 2), the irrigation method, which is determined on the basis of the predicted ET_c_, has the characteristics of many times a small amount of irrigation, can accurately regulate soil moisture at appropriate interval, thus promoting the growth of the root system, and facilitating the absorption of the resources by the root system^72,73^. Therefore, it improves the transfer of nutrients and photosynthesis products to reproductive organs^73,74^and consequently increases the number of fruits^75^. In our experiments, the increase in yield with increasing number of irrigations was mainly due to an increase in the number of fruits^75^ as well as an increase in the weight of individual fruits (Fig. 8), which is in agreement with the results of previous studies on other crops, such as tomato^63,74,76^, bell pepper^77^and melon^78^.

In addition, Table 7. shows the comparison of the quality and yield of dried fruit of *Lycium barbarum* of the PCA-based optimal treatment W0FM in this study with those of the control treatment CK, the optimal I_2_N_2_treatments (with an irrigation volume of 2563 m^3^ ha^−1^ and N application of 225 kg ha^−1^) of Ma et al.^13^ and Yin et al.^14^ in the same study area, and the optimal W1N2 combination (with an irrigation volume ranging from 315.4 to 374.3 mm and N application ranging from 300.0 to 308.3 kg ha^−1^) of Tian et al.^15^ and Gao et al.^16,17^ in different study areas. Overall, the quality and yield of dried fruit of *Lycium barbarum* under the optimum W0FM treatment in this study were mostly greater than those under the CK and I_2_N_2_ treatments in the same study area, and the amount of change in the quality of dried fruit of *Lycium barbarum* (except proteins and polysaccharides) and yield was the lowest over two consecutive years. This occurred beacause during the two consecutive years of experimentation in this study, there was more rainfall in 2021 (171.9 mm) and less rainfall in 2022 (85.4 mm), and the irrigation in the control treatment (CK) and the I_2_N_2_ treatment, which was based on the quota irrigation method, was a static irrigation regime that was not able to adapt to climate change and effectively cope with climatic drought, thus resulting in a reduction in the quality of *Lycium barbarum*. On the other hand, the optimal W0FH treatment under the dynamic irrigation regime based on the predicted ET_c_ control of irrigation volume maintained stable *Lycium barbarum* quality and yield by maximising the use of rainfall and effectively coping with climatic drought. The quality and yield of dried fruit of *Lycium barbarum* under the optimal W0FM treatment in this study were weakly comparable to those under the optimal W1N2 treatment in the different study areas, and the results were for reference only.

**Table 7.** Comparison of the quality indexes and yield indexes of the dried fruit of *Lycium barbarum* in the optimal W0FM treatment in this study with those in the control CK treatment and the optimal treatments in other experiments.

Test indicators		Test site and test treatment			
		Ningxia, China			Gansu, China
		This study		Ma et al.^13^, Yin et al.^14^	Tian et al.^15^, Gao et al.^16,17^
		W0FM	CK	I_2_N_2_	W1N2
Protein (g 100g^−1^)	2021	13.00	14.35	10.97	12.05
	2022	13.67	14.22	10.83	13.51
	Variation	0.67	−0.13	−0.14	1.47
Fat (g 100g^−1^)	2021	1.67	1.62	1.97	2.07
	2022	1.60	1.45	1.74	2.19
	Variation	−0.07	−0.17	−0.23	0.13
Polysaccharide (g 100g^−1^)	2021	5.00	4.92	5.25	5.88
	2022	5.30	4.81	5.25	5.60
	Variation	0.30	−0.11	0.00	−0.28
Total Sugar (g 100g^−1^)	2021	51.07	52.54	49.80	52.04
	2022	52.07	47.65	36.15	53.22
	Variation	1.00	−4.89	−13.65	1.19
Beataine (g 100g^−1^)	2021	0.79	0.72	0.57	/
	2022	0.84	0.69	0.78	/
	Variation	0.05	−0.04	0.21	/
100-grian weigh tof dry fruit (g)	2021	18.91	16.22	17.24	20.76
	2022	18.34	14.51	16.54	20.46
	Variation	−0.62	−1.71	−0.7	−0.30
Dried fruit yield (kg ha^−1^)	2021	2573.15	1471.52	2320.94	2530.04
	2022	2583.11	1594.77	2391.73	2716.14
	Variation	9.95	−146.7	70.79	186.10

## Conclusion

The optimal regulation of soil wate-fertiliser coupling under the irrigation volume determined by the predicted ET_c_ increased the water and nutrient uptake by the crop root system so that the water-fertiliser worked synergistically to increase the ability of the *Lycium barbarum* plant to carry out photosynthesis, which was conducive to the accumulation of dry matter mass in the plant and the increase in fruit quality and yield to improve quality and efficiency. The main conclusions are as follows:

(1) During the growth period of *Lycium barbarum*, except the spring branch growth period, the chlorophyll content of *Lycium barbarum* leaves was extremely significantly affected by the irrigation volume, which was determined on the basis of the predicted ET_c_, fertiliser application and their interactions. The irrigation volume, which was determined on the basis of the predicted ET_c,_ had the greatest effect on the daily changes in the photosynthetic indexes of *Lycium barbarum* leaves, followed by fertiliser application and, finally, the water-fertiliser interaction. In addition to betaine, the yield and other dry fruit qualities of *Lycium barbarum* were significantly affected by the irrigation volume, which was determined on the basis of the predicted ET_c_ and fertiliser application, and the yield of *Lycium barbarum* was also significantly affected by water-fertiliser interactions.

(2) Taking photosynthesis, quality and yield of *Lycium barbarum* into account, the comprehensive evaluation based on PCA revealed that the optimal combination of water and fertilisation under the irrigation volume determined on the basis of the predicted ET_c_ was W0FM for both years; i.e., the quality and yield of *Lycium barbarum* with an irrigation level of 100% ET_c_ (254.2 mm in 2021, 309.4 mm in 2022) and a fertiliser level of FM (fertiliser application: N-P_2_O_5_-K_2_O of 315-82-135 kg ha^−1^) were better. This study provides a basis for rational water and fertiliser management of *Lycium barbarum* production in the arid zone of Northwest China, especially for the comprehensive optimisation of crop photosynthesis, quality and yield.

## Data Availability

The datasets used and analysed during the current study available from the corresponding author on reasonable request.
